# mCSM-lig: quantifying the effects of mutations on protein-small molecule affinity in genetic disease and emergence of drug resistance

**DOI:** 10.1038/srep29575

**Published:** 2016-07-07

**Authors:** Douglas E. V. Pires, Tom L. Blundell, David B. Ascher

**Affiliations:** 1 Department of Biochemistry, Sanger Building, University of Cambridge, 80 Tennis Court Road, Cambridge, CB2 1GA, UK; 2 Centro de Pesquisas René Rachou, Fundação Oswaldo Cruz, Avenida Augusto de Lima 1715, Belo Horizonte, 30190-002, Brazil

## Abstract

The ability to predict how a mutation affects ligand binding is an essential step in understanding, anticipating and improving the design of new treatments for drug resistance, and in understanding genetic diseases. Here we present mCSM-lig, a structure-guided computational approach for quantifying the effects of single-point missense mutations on affinities of small molecules for proteins. mCSM-lig uses graph-based signatures to represent the wild-type environment of mutations, and small-molecule chemical features and changes in protein stability as evidence to train a predictive model using a representative set of protein-ligand complexes from the Platinum database. We show our method provides a very good correlation with experimental data (up to ρ = 0.67) and is effective in predicting a range of chemotherapeutic, antiviral and antibiotic resistance mutations, providing useful insights for genotypic screening and to guide drug development. mCSM-lig also provides insights into understanding Mendelian disease mutations and as a tool for guiding protein design. mCSM-lig is freely available as a web server at http://structure.bioc.cam.ac.uk/mcsm_lig.

The wealth of information arising from second-generation genome sequencing is demonstrating that future responses to two major areas of human health and disease will very often depend on understanding the effects of missense mutations on ligand binding to proteins. In many genetic diseases (Mendelian disorders), for example Alkaptonuria^1^,^2^, mutations are observed to affect the binding of substrates or ligands in active sites. In a similar way drug resistance, which is frequently due to the effects of mutations on drug recognition of protein targets^3^, is of growing significance not only to developing nations as a result of the use of antibiotics in tuberculosis, malaria and other infectious diseases, but also throughout the world due to the overuse of drugs in fast evolving cancers and antibiotics for infections, which will also have huge impacts on safety in surgery^4^. Both genetic disease and drug resistance require initial characterisation of changes in the individual human or pathogen genome sequence, allowing us to prioritise treatment strategies, and then use of this information to design better drugs as part of a new personalised or precision medicine.

The recent explosion in high-throughput sequencing has provided a unique opportunity to address these problems, but determining the effects of missense mutations on protein-ligand interactions in a high-throughput manner remains a real challenge. Experimental approaches, while not rapid or cheap, have allowed direct measurement of the impact of these mutations, but they are poorly equipped to tackle the vast amounts of data being generated not only from individuals with a multitude of genome variations that may give rise to similar genetic disease but also from fast evolving genomes of pathogens and tumours. This, together with the lack of a comprehensive repository linking effects of mutations in proteins with experimentally defined structures of protein-ligand complexes, has hindered the development of quick and effective computational approaches. An accurate computational tool that allows rapid evaluation of the potential effects of a mutation would shed light on anticipating and understanding mutations that give rise to both genetic disease and drug resistance.

Over the past two decades, several efforts in establishing comprehensive databases linking experimentally measured effects of mutations to protein structures^5^,^6^ have supported the development of computational methods to assess the multitude of impacts of a mutation on structure and function. Most early methods focused on predicting how mutations affect protein stability^7^,^8^,^9^,^10^,^11^,^12^, and more recently the change in interaction affinity, including protein-protein^11^,^13^,^14^,^15^ and protein-nucleic acid^11^,^13^ binding.

Many attempts to predict and model the effects of mutations on protein-small molecule interactions from a structural perspective have been employed, however with limited success or applicability. These include computationally intensive approaches such as the use of force fields for direct estimation of free energies of binding^7^,^16^ and molecular dynamics^17^,^18^,^19^. Most of these approaches rely on modelling a mutation on a wild-type structure^20^ and/or docking of the small molecule^21^,^22^,^23^,^24^, which will likely provide useful insight. However, while local changes may be predicted, allosteric changes are much more challenging to anticipate and encompass, such as those reported to alter the protein dynamics of NS5A^25^. A recent report has provided an alternative to these, by using pair-potentials (log odds) to predict whether a given mutation enhanced or diminished interactions in a protein complex based on the frequency of a given residue in proteins binding the considered ligand class^13^. This has shown interesting results for protein-protein, protein-DNA and protein-ion interactions, but limited success on protein-small molecule complexes.

Our group has previously used the concept of graph-based structural signatures to model several different molecular phenomena. In a broad sense, structural signatures represent sets of invariant features that unequivocally describe the similarity between entities. By using a graph-based representation of a wild-type protein structure combined with implicit information on the change the mutation would introduce into the structure, we were able to predict accurately and scalably the effects of mutations on protein stability^11^, protein-protein affinity^11^,^26^ and protein-nucleic acid affinity^11^ by training predictive models using machine learning methods. One of the advantages of this approach has been that it does not require modelling the mutant structure and is not limited to local changes. Analysis of disease-causing mutations has shown that a range of these different structural effects is responsible for the phenotype^1^,^2^,^27^,^28^,^29^. We have also recently used these graph-based signatures to model small molecule chemistry^30^,^31^, enabling the prediction of binding affinity, pharmacokinetic and toxicological properties. A full understanding of the link between genotype and phenotype, however, has been limited by the absence of a comparable measure of the effects on small molecule binding.

The complexity of small molecule chemistry and binding interactions has hindered the development of methods to assess the effects of mutations. This is compounded by the multitude of effects on stability and interplay with partners that mutations trigger in proteins. However, obtaining mathematical representations of the effects of mutations and then extracting relevant information from them to build predictive models are major challenges to be addressed. Together our previous methods provide the foundation for assessing ligand-affinity changes upon mutations as they enable us to model the protein and small-molecule chemistry through their respective graph-based signatures, as well as modelling other effects that can influence ligand binding, including stability changes.

One of the main limitations of the development of novel machine learning methods is the availability of high quality data that can be used for training. The degree of generalization of a predictive model is directly related to the confidence, variability and quality of the data set used. Even though a considerable amount of information on the effects of mutations has been accumulated over the years and organized in relational databases, until very recently no comprehensive, large-scale database compiling effects of mutations on the affinity of protein-small molecule complexes has been available. In order to fill this gap, we have assembled the Platinum database^32^, a manually curated, large-scale relational database that for the first time associates experimentally measured effects of mutations on small-molecule binding for solved complexes deposited in the Protein Data Bank (http://structure.bioc.cam.ac.uk/platinum). Studies of protein-ligand interactions show that small changes in experimental conditions can have large effects on the affinity. In Platinum we therefore required ligand-affinity measurements for wildtype and mutant proteins to be performed by the same group under the same conditions. Platinum currently comprises just over 1,000 mutations spanning 250 unique protein-ligand complexes and forming a very diverse set.

The availability of the Platinum database now allows us to propose a structure-guided, computational approach to assess directly and quantify the effects of mutations on ligand binding, taking into account the multitude of effects by which mutations trigger changes in protein structures and how they recognise small molecules. Here we describe mCSM-lig, a method that provides a reliable, scalable way to predict and characterize single-point mutations affecting small molecule binding. The new method has applications in understanding at a molecular level the emergence of drug resistance across any pathogen, including viruses, bacteria, and cancer, for understanding genetic diseases, and as a tool in protein engineering.

## Results

### Computational Approach

The approach uses a graph-based structural signature for protein-ligand complexes that models both the topology and physicochemical properties of the interactions and architecture of the wild-type protein-ligand complex by representing atoms as nodes and interactions between them as edges. Atoms are labelled based on pharmacophore types, as described in the Methods Section. From this representation, distance patterns between atoms categorized by their properties are summarized in concise signatures as cumulative distributions, encompassing the frequency of atom pairs per distance, per atom type. A more detailed description of how the signatures are calculated is available as Supplementary Material.

Complementary information also integrated in the signatures includes predicted changes in protein stability to account for changes in flexibility of the binding site (correlation of ρ = 0.188 with experimental data), the type of the ligand in the complex (its molecular properties, e.g., molecular weight, logP, surface area and others) (correlation of ρ = 0.255 with experimental data), the significance of the local/direct effect of the mutation (denoted by the distance from mutated residue to ligand), residue depth^33^, and affinity of the wild-type complex. These information together with the graph-based features are then used as evidence for machine learning methods. The complete list of features used is available in the Methods.

**Figure 1:**
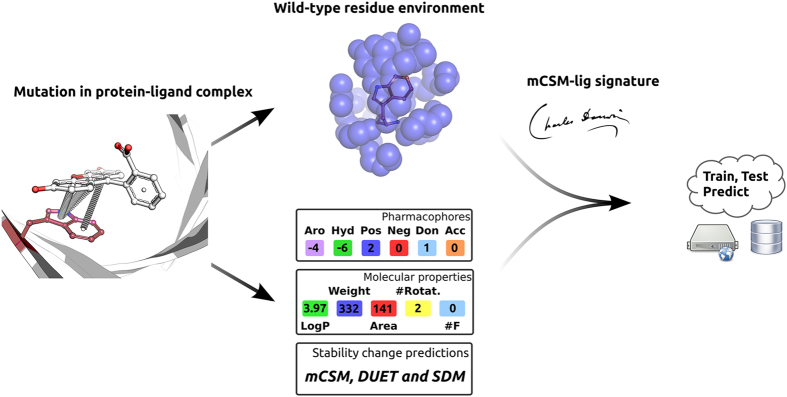
Predicting the impacts of mutations on protein-ligand affinities using mCSM-lig. This workflow highlights important steps in the methodology and how the main components of the signatures are computed. Here we use as an example the engineered lipocalin FluA binding to fluorescein (PDB ID: 1N0S, ligand ID: FLU), considering the mutation W129H. Given a mutation site in a wild-type protein, its structural environment is extracted and the distance patterns among the atoms summarized in the mCSM-lig signature. To take into account the change in atom types due to the mutation, a pharmacophore count is performed for the wildtype and mutant residue. The changes in pharmacophore count, ligand physicochemical properties and estimations of protein stability are then appended into the signature, which is used to train/test predictive models. This figure was created using yED, 3.14.3 (https://www.yworks.com/products/yed).

This information, together with the experimentally measured effect of the mutation, can then be used to train and test prediction models. Fig. 1 summarizes the main components and steps of the mCSM-lig workflow.

### Quantitative assessment of affinity changes upon mutation

In order to assess the performance of our methodology in quantifying the effects of mutations on protein-ligand affinity (a regression task) we have evaluated each component of the signatures both separately and together, in an attempt to understand better the relative importance of each in our structural signature. For regression models, the goal was the affinity-fold change of a given missense single-point in logarithm scale:


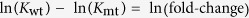


where *K*_wt_ denotes the affinity of the wild-type proten-ligand complex and denotes *K*_mt_ the affinity of the mutant complex. Table 1 shows the performance of mCSM-lig and its main components separately in regression tasks. The method achieved a Pearson’s correlation coefficient of ρ = 0.628 on 10-fold cross validation with a standard error of 2.059, using a Gaussian Process model.

The standard errors in mCSM-lig seem proportional to the interval of experimental affinity changes used in training. Most of the large prediction errors are associated with extreme values, and are often a result of multiple severe molecular effects. These are usually difficult data points for machine learning methods to predict, and tend to be underestimated, however it is important to highlight that these mutations are still correctly identified as either significantly increasing or decreasing binding affinity.

The main feature responsible for the performance of mCSM-lig is the graph-based signature that encodes both geometry and physicochemical properties internal to the protein and between protein and ligand. The best performing signature obtained was the combination of the described features in Table 1. This model was also evaluated under different validation schemes where the performance obtained proved to be maintained consistently, as shown in Table S1. This strengthens our confidence in the method’s predictive and generalization capabilities.

We have also evaluated mCSM-lig in classification tasks where mutations were labelled as causing a “decrease” or “increase” in ligand affinity. 505 and 259 mutations were assigned to these two classes, respectively. mCSM-lig achieved an accuracy of 0.761 on 10-fold cross validation, with a precision of 0.755 and Area Under ROC Curve (AUC) of nearly 0.8, as shown in Table S2. For these experiments all components seem to play important roles in separating the mutation classes.

### Outlier Analysis

**Figure 2:**
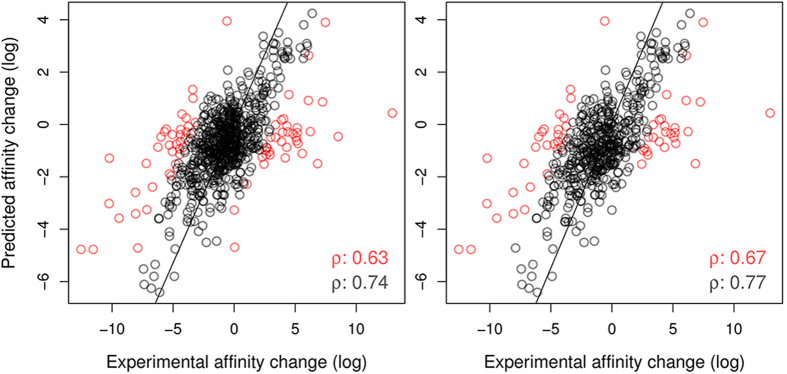
Regression plot between experimental and predicted effects of mutation on ligand affinity on the full data set (763 mutations, left graph) and on mutated residues close to the ligand (545 mutations ≤5 Å, right graph). mCSM-lig achieved a Pearson correlation coefficient of ρ = 0.627 over the entire data set and ρ = 0.674 for those mutations close to the ligand, with this correlation improving to ρ = 0.737 and ρ = 0.769 respectively after 10% outlier removal. Outliers are depicted in red.

In order to understand better how the performance of mCSM-lig is distributed across the data set, we have evaluated its performance after outlier removal, considering several thresholds as shown in Table 2. The correlation coefficient of mCSM-lig increases considerably to ρ = 0.699 after just 5% of outliers are removed, getting to ρ = 0.737 after 10%. This increase in performance is depicted in Fig. 2, left graph.

As expected, predicting direct effects on ligand binding, i.e. the effects of mutations directly in contact with the ligand, is a task where mCSM-lig performs best. Considering the subset of mutations within 5 Å of the ligand (545 mutations) the correlation coefficient obtained was ρ = 0.674, increasing to ρ = 0.769 after 10% outlier removal, as depicted in Fig. 2, right graph.

The outliers identified seem to form two clusters with extreme experimental values (highly decreasing/increasing affinity), as shown in Figure S1A and S1B which are expected to be the most difficult cases for machine learning methods, given the distribution of experimental values. For those mutations with extreme experimental values, mCSM-lig correctly classified approximately 85% of the mutations as either improving or degrading ligand affinity, highlighting that, while they may be outliers, mCSM-lig is strong in predicting the nature of effects that mutations exert.

### mCSM-lig Webserver

In order to provide mCSM-lig functionalities to the scientific community and increase reproducibility we have implemented a user-friendly webserver freely available at http://structure.bioc.cam.ac.uk/mcsm_lig. The server front-end was built using Bootstrap framework version 2.0, while the back-end was built in Python via the Flask framework (version 0.10.1), running on a Linux server. It allows users to upload protein-ligand complexes (in PDB format) and inform mutations for which the impact on ligand affinity can be predicted.

### Drug-Resistance: mCSM-lig Identifies BCR-ABL Mutations Leading to Chemotherapeutic Resistance

Within the Platinum database there were no binding data available with Imatinib (Ligand ID: STI) as a ligand, and only three characterised mutations of the tyrosine kinase ABL, of which only one fit the criteria of the training set, but was excluded from training mCSM-lig. We therefore used this unbiased case example to predict the effects of 83 BCR-ABL mutations identified in clinical samples from patients reported to be resistant to Imatinib^34^ (which are well spread over the protein, as depicted in Figure S2A). In this scenario, we compared mCSM-lig predictions for the drug and for the natural ligand, ATP. A mutation was considered to be leading to resistance when its impact on binding of the drug was predicted to be greater than that on the natural ligand. The PDB IDs 2HYY and 2G2I were used as wild-type structures of the BCR-ABL/Imatinib and BCR-ABL/ATP complexes, respectively. Since the 2G2I structure had BCR-ABL bound to ADP, the additional phosphate of ATP was modelled in using PyMOL.

The predictions and fold-change ratios obtained from this analysis are shown in Table S3. Values greater than one denote a preference change towards the natural ligand. By using 1.2 as a ratio threshold (chosen via systematic evaluation and selecting the best performing cutoff), mCSM-lig was able to identify correctly 83% (69 out of 83) of the resistance mutations, a result that is compatible with the performance of the method during cross validation for classification experiments (Table S2).

One of the sensitivities against which we wished to evaluate mCSM-Lig was the ability to differentiate resistance-mutation profiles for different drugs binding to the same site. In response to the development of resistance to Imatinib, a number of second generation drugs, including Nilotinib (Ligand ID: NIL) and Dasatinib (Ligand ID: 1N1), were developed that had a higher genetic barrier to the development of resistance. In the TKI-Resistance Mutations genetic testing database^35^, 24 mutations were tested based on *in vitro* potency and shown to be resistant to Nilotinib (Figure S2B) while eight were resistant to Dasatinib (Figure S2C). Using mCSM-Lig, we evaluated the effects of these mutations on the relative affinities of BCR-ABL for the respective drugs (PDB ID: 3CS9 and 2GQG for Nilotinib and Dasatinib respectively) and ATP (from above). mCSM-lig was able to predict over 75% of resistance mutations correctly (Table S3), also using 1.2 as a ratio threshold. This demonstrates the potential for mCSM-lig to explore and predict the resistance profiles expected for different molecules.

### Antiviral Resistance Mutations: mCSM-lig Identifies Non-nucleoside Inhibitors Resistance Mutations in HIV Reverse Transcriptase

The relatively rapid emergence of resistance mutations against the first generation non-nucleoside inhibitors to HIV reverse transcriptase led to the design of second generation drugs (including Efavirenz; Ligand ID: EFZ) with a higher genetic barrier to the development of resistance and third generation inhibitors (including Rilpivirine; Ligand ID: T27) with increased efficacy against drug resistant mutants^36^.

The International Antiviral Society-USE (IAS-USA) and Stanford HIV drug databases were used to identify the most common clinically observed resistance mutations in HIV-1 reverse transcriptase^36^. This identified 23 mutations in HIV-1 reverse transcriptase that led to reduced susceptibility or virological response against Efacirenz (Figure S2D) and 25 mutations showing reduced susceptibility against Rilpivirine (Figure S2E). mCSM-Lig has been used to predict the effects of these mutations on the binding affinities of Efacirenz and Rilpivirine, using the available X-ray crystal structures (PDB ID: 1FK9 and 3MEE respectively). Correlation with the characterised sensitivities of the mutations against the respective drugs showed that mCSM-Lig was able to identify accurately mutations that respond to or are resistant against Efacirenz and Rilpivirine, with 82.6% and 80% sensitivity respectively (Table S4, using a threshold of −0.3, which was chosen via systematic evaluation and selecting the best performing cutoff).

### Mendelian Diseases: mCSM-lig Characterises the Effects of Mutations in Alkaptonuria

Previously we have shown that mutations in Alkaptonuria result in the inactivation of homogentisate 1,2-dioxygenase (HGD) by several mechanisms, including through destabilisation of the protomer, by preventing formation of the active hexamer, or by interfering with its active site leading to alteration of substrate recognition or catalytic activity^1^,^2^. At the time we had no reliable computational approach to predicting the effects of a given mutation upon substrate binding. However, we have now been able to investigate this using mCSM-lig, so providing an example of its application to understanding the effects of mutations that modulate small molecule binding in genetic diseases. In the HGD mutation database there are 12 mutations assigned as affecting the active site (Figure S2F). Using the crystal structure of the *Pseudomonas putida* HGD (PDB ID: 4AQ6) in complex with its substrate homogentisic acid (Ligand ID: OMD) the ligand pose was transferred into the structure of the human HGD (PDB ID: 1EY2) via structural alignment and the modelled complex minimized in Maestro (Schrodinger, New York, NY). mCSM-lig was used to predict the affects of these mutations upon the affinity for the ligand, predicting them to lead to a reduction in affinity for homogentisic acid by HGD (average change −0.918 log(fold change)). Comparing the predicted effects of these mutations against those of 86 missense non-pathogenic polymorphisms from the 1000 Genome Database^37^, we conclude that the active site disease mutations lead to a significantly larger decrease in affinity for homogentisic acid (average change polymorphisms −0.315 log(fold change); p = 0.0046) (Table S5).

### Mendelian Diseases: mCSM-lig Characterises the Effects of Mutations in Millers Syndrome

Millers syndrome is a genetic developmental disease resulting from a loss of the mitochondrial protein dihydroorotate dehydrogenase, part of the UMP biosynthesis pathway. Nine mutations in dihydroorotate dehydrogenase have been associated with the development of Millers Syndrome^38^. Several X-ray crystal structures of dihydroorotate dehydrogenase have been solved in the presence of its substrate, orotic acid (Ligand ID: ORO), and its cofactor, riboflavin monophosphate (Ligand ID: FMN). mCSM-Lig was used to assess the effects of these mutations on binding affinities of FMN and ORO (PDB ID: 4LS0). The disease-causing mutations were predicted to lead to a loss of affinity for small molecules by an average of −0.576 log(fold change). Comparing these mutations to 57 missense polymorphisms present in the 1000 Genome Database^37^, we observed that the disease associated mutations lead to a significant decrease in affinity for the two ligands (average change polymorphisms −0.258 log(fold change); p = 0.0428) (Table S5).

## Discussion

Here we have described mCSM-lig, a new structure-based computational approach for predicting the effects of missense mutations on small-molecule protein binding affinity. Our method is built on the concept of graph-based structural signatures, demonstrating that they provide a concise and effective way to represent both geometry and physicochemical properties of protein-ligand interactions. We show our method not only performed well in quantifying and assessing directly the effects of mutations but was also validated successfully in blind tests for identifying resistance mutations in different systems, characterizing mutations in genetic diseases and as a tool for optimizing ligand binding.

By using mCSM-lig we were able to identify correctly mutations giving rise to resistance against first and second-generation tyrosine kinase inhibitors in chemotherapy, antiviral resistance mutations against second and third generation non-nucleoside reverse transcriptase inhibitors and antibiotic resistance mutations against the β-lactam antibiotics, including penicillin (Supplementary Information). Looking at other well studied systems such as the neuraminidase inhibitors, mCSM-Lig was able to identify mutations previously characterised as leading to reduced Oseltamivir and Zanamivir activity, including E119V and H274Y^39^. This suggests that mCSM-lig is a very useful tool in the investigation of resistance mutations in different organisms and protein systems, and potentially early in the drug discovery process in order to help guide medicinal chemistry efforts.

We were also able to compare pathogenic mutations in two different genetic diseases, Millers Syndrome and active site mutations in Alkaptonuria^1^,^2^, and show that these mutations presented significantly larger effects on ligand binding compared to neutral mutations Thus, mCSM-lig is also helpful in evaluating the molecular effects of missense mutations in genetic diseases. The ability of mCSM-lig to predict accurately the effects of mutations in the engineered fluorescein-binding protein FluA (Supplementary Information) suggests that it may be a valuable tool in the intelligent design of proteins with novel binding properties.

It is important to acknowledge that the effects of mutations on ligand binding in both genetic diseases and drug resistance are not always orthosteric. As we have previously observed in both Alkaptonuria and in resistance to first and second line *Mycobacterium tuberculosis* antimicrobials, mutations may disrupt ligand binding through classical allostery, by binding at sites at a distance from the ligand, or by disrupting interfaces in functional homo- or hetero-oligomers^1^,^2^.

We believe mCSM-lig for the first time will allow inquiries regarding effects of mutations on small molecule binding affinity, not only in an unprecedented scale but also at atomic resolution, which should contribute to a better understanding of the driving forces of protein-ligand molecular recognition, the role of mutations in genetic disease and the emergence of drug resistance, as well as guide further experimental investigations, including the design of novel proteins with tuned ligand affinity and specificity. A user-friendly web server implementing mCSM-lig functionality was implemented and is freely available at http://structure.bioc.cam.ac.uk/mcsm_lig.

## Methods

### Data Sets

In order to use machine learning to train a predictive model, we required a high quality set of experimental data on the impacts of mutations upon protein affinity. We have selected a subset of single-point mutations affecting ligand binding from the Platinum database^32^ and these were used to train and test mCSM-lig. Ligands broken in multiple IDs were removed as well as Cα model structures, resulting in 763 mutations on over 200 protein-ligand complexes, which were filtered and standardized using PDBest^40^. Figures S3A-F shows the main properties of the 177 unique ligands that form this data set, demonstrating a good diversity both in terms of size and physicochemical aspects. In addition, as shown in Figures S3G and S3H, 75% of the mutated residues considered are directly in contact with the ligand (distance less than 5 Å). The selected mutations are also diverse in terms of their effects, as depicted in Figure S1C, which highlights the distribution of the difference in experimental affinities between wild-type and mutant protein-ligand complex (2/3 resulting in a reduced affinity).

### Signature Components

The two main components of the signatures used in mCSM-lig to train the predictive model are the graph-based cumulative distribution of distances^11^ and a series of molecular descriptors designed to encompass the residue environment of mutated residue, the effects of the mutation in potential interactions as well as ligand properties. These descriptors used are: predicted changes in protein stability^8^,^11^; residue depth calculation^33^; the distance from mutated residue to ligand in angstroms; the pharmacophore change, which is the difference in atom numbers per class between wild-type and mutant residues considering eight classes (Hydrophobic, Positively charged, negatively charged, Hydrogen Acceptor, Hydrogen Donor, Aromatic, Sulphur and Neutral); Wild-type affinity of the protein-ligand complex; and ligand properties calculated by the Python RDKit toolkit based on SMILES including molecular weight and number of heavy atoms, LogP, numbers of hydrogen bond acceptors and donors, rotatable bonds, number of rings, surface area (Labute and TPSA).

### Machine learning methods

To obtain a predictive regression model based on the mCSM-lig workflow, a Gaussian Process^41^ was trained, based on the implementation available through the Weka toolkit. For classification tasks, the Random Forest algorithm was used. The final regression model was trained and evaluated under different schemes, ranging from 5-fold, 10-fold and leave-one-out cross validations, presenting comparable performances.

### Evaluation metrics

Classification models were evaluated based on metrics, including the Area Under the ROC curve (AUC), precision (tp/(tp+fp)) and accuracy ((tp+tn)/(tp+fp+tn+fn)). Regression models were evaluated based on Pearson’s correlation coefficient (ρ) and standard error. In order to assess the method’s performance on the majority of the data set minimizing large effects arising from a few data points, correlations are evaluated before and after 10% outlier removal.

## Additional Information

**How to cite this article**: Pires, D. E. V. *et al.* mCSM-lig: quantifying the effects of mutations on protein-small molecule affinity in genetic disease and emergence of drug resistance. *Sci. Rep.* **6**, 29575; doi: 10.1038/srep29575 (2016).

## Supplementary Material

Supplementary Information

## Figures and Tables

**Table 1:** Performance of the computational approach on regression tasks.

Feature	Ρ	Std. Error
Pharmacophore difference	0.181	2.604
Stability prediction	0.188	2.584
Ligand properties	0.255	2.545
Graph-based signatures	0.569	2.167
mCSM-lig	0.628	2.059

The mCSM-lig denotes a combination of the described features and presents a significant improvement in performance in comparison with either individual feature. Performance assessed on 10-fold cross validation using a Gaussian Process.

**Table 2:** Performance of mCSM-lig after outlier removal for the complete set of mutations and for those in contact with the ligand.

% of the data set used	Full data set (#763)	Distance to ligand ≤5 Å (#545)
100%	ρ = 0.627	ρ = 0.674
95%	ρ = 0.699	ρ = 0.729
90%	ρ = 0.737	ρ = 0.769
80%	ρ = 0.801	ρ = 0.824
