# Supplementary material for: mCSM-lig: quantifying the effects of mutations on protein-small molecule affinity in genetic disease and emergence of drug resistance
Source: Sci Rep. 2016 Jul 7;6:29575. doi: 10.1038/srep29575 (PMC4935856; doi:10.1038/srep29575)
Supplement: Supplementary Information [file srep29575-s1.pdf]

Supplementary Material

mCSM-lig: quantifying the effects of mutations on protein-small molecule affinity in genetic disease and emergence of drug resistance

Douglas E. V. Pires[a,b], Tom L. Blundell[a], David B. Ascher[a]

[a] Department of Biochemistry, Sanger Building, University of Cambridge, 80 Tennis Court Road, Cambridge, CB2 1GA, UK.

[b] Centro de Pesquisas René Rachou, Fundação Oswaldo Cruz, Avenida Augusto de Lima 1715, Belo Horizonte, 30190-002, Brazil

Correspondence should be addressed to D.B.A: Tel: +44 1223766033; Fax: +44 1223766002; mail: dascher@svi.edu.au or da382@cam.ac.uk; Correspondence may also be addressed to douglas.pires@cpqrr.fiocruz.br or tom@cryst.bioc.cam.ac.uk

Supplementary Information

mCSM-lig - Graph-based signature calculation: The main component present in the mCSM-lig signatures is derived from the structural features defined by the CSM algorithm, originally proposed by (1) and adapted to represent a residue environment in (2). The mCSM-lig algorithm defines a series of graph-based structural signatures which correspond to cumulative distributions of atom pair distances, by type, used as evidence to train and test predictive models. Algorithm 1 provides a description of the procedure for calculating the signatures.

Algorithm 1: Procedure for signature calculation used in mCSM-lig.

Algorithm 1 mutation Cutoff Scanning Matrix (mCSM) calculation

```
function mCSM-LIG(MutationSet, AtomClass, D_MIN, D_MAX, D_STEP)
    for all mutation i ∈ (MutationSet) do
        residue_environment = extractResidueEnvironment(mutation)
        j = 0
        distMatrix ← calculateAtomicPairwiseDist(residue_environment)
        for dist ← D_MIN; to D_MAX; step D_STEP do
            for all class ∈ (AtomClass) do
                mCSM-lig[i][j] ← getFrequency(distMatrix, dist, class)
                j++
        add_predicted_stability_changes(mCSM-lig[i])
        add_ligand_properties(mCSM-lig[i])
        add_pharmacophores_changes(mCSM-lig[i])
        add_distance_mutated_res_to_ligand(mCSM-lig[i])
        add_affinity_wildtype_complex(mCSM-lig[i])
    return mCSM-lig
```

Antibacterial Resistance Mutations: mCSM-lig Identifies Mutations in Penicillin Binding Protein Leading to Penicillin Resistance: Resistance against Penicillin (Ligand ID: PNV), the first of the β-lactam antibiotics, arose within a decade of its first use. While still widely used today,

their extensive use has led to the spread of resistance. A number of experimental studies have looked at changes in the Penicillin Binding Proteins (PBP) and its relation to the development of this resistance. For example, Yamachika and collegues (3) identified a number of point mutations in *Escherichia coli* PBP2 that led to resistance. Building a homology model of *E. coli* PBP2 using homologous experimental structures (PDB ID's: 1MWU, 1PMD, 3OC2, 3PBQ, 3VSL, 4YE5), we used AutoDock to generate a model of E. coli PBP2 in complex with Penicillin and Carbapenem (Ligand ID: MER). Docking was guided by experimental co-crystal structures, and poses were consistent with the literature. mCSM-lig predicted that 75% of the mutations would result in a decrease in binding affinity of the β-lactam antibiotics.

β-lactam antibiotic resistance in Neisseria gonorrhoeae, in particular to the last remaining first line treatments Ceftriaxone and Cefixime, has become a significant problem. This resistance is primarily due to changes in PBP2. We used AutoDock to dock Penicillin and Cefixime (Ligand ID: C04) into the experimental structure of PBP2 (PDB ID: 3EQU). We predicted the effect of 22 point mutations on the binding affinity of penicillin (Figure S2G) and cefixime (Figure S2H) using mCSM-lig. These mutations had been previously investigated experimentally. mCSM-lig correctly identified whether a given mutation would lead to an increased MIC and resistance or not for both Penicillin (17/22 - 77%) and cefixime (18/22 - 82%) (Table S6). We also observed that mCSM-lig was able to correctly identify mutations that displayed altered sensitivity levels between Penicillin and cefixime.

Protein design: mCSM-lig Can Identify Mutations that Degrade or Improve Ligand Recognition: FluA is an engineered protein selected through random mutagenesis of the bilin-binding protein for its ability to bind to fluorescein(4). Subsequent efforts have focused on trying to improve the affinity of FluA for fluorescein (Ligand ID: FLU). Predicting advantageous mutations is a major challenge due to the higher proportion of reported deleterious mutations. Most methods in the literature are biased, to differing extents, towards classes of mutations interfering with fluorescein and anticalins, but neither was included in Platinum or the data used to train mCSM-Lig, and were therefore used to evaluate the ability of mCSM-Lig to predict mutations improving ligand binding affinity and its usefulness in protein engineering approaches.

Vopel and colleagues designed and tested 11 mutants in the ligand binding pocket of FluA for their ability to bind to fluorescein(5) (Figure S2I). Exploiting knowledge of the X-ray crystal structure of FluA in complex with fluorescein (PDB ID 1N0S), we used mCSM-Lig to predict the effects of these mutations. Comparing the experimentally measured changes in binding affinity to those predicted by mCSM-Lig yielded a correlation of 0.69, consistent with the overall performance of mCSM-Lig.

Supplementary Figures

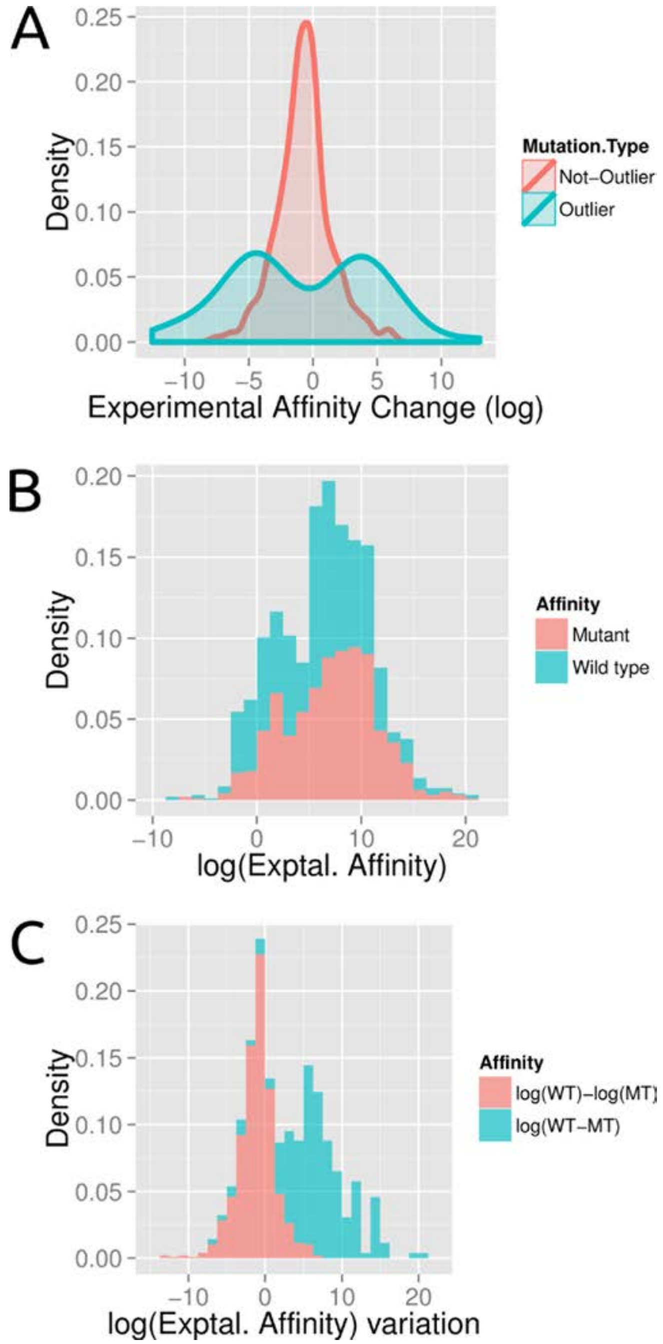

Figure S1. Distribution of experimental affinity changes (log) for outliers and the remaining mutations. Histogram of experimental affinities (log scale) for wild-type and mutant protein-ligand complexes (A) and the affinity variation (B). Out of the 763 mutations in this dataset, 505 reduced protein-ligand affinity (C).

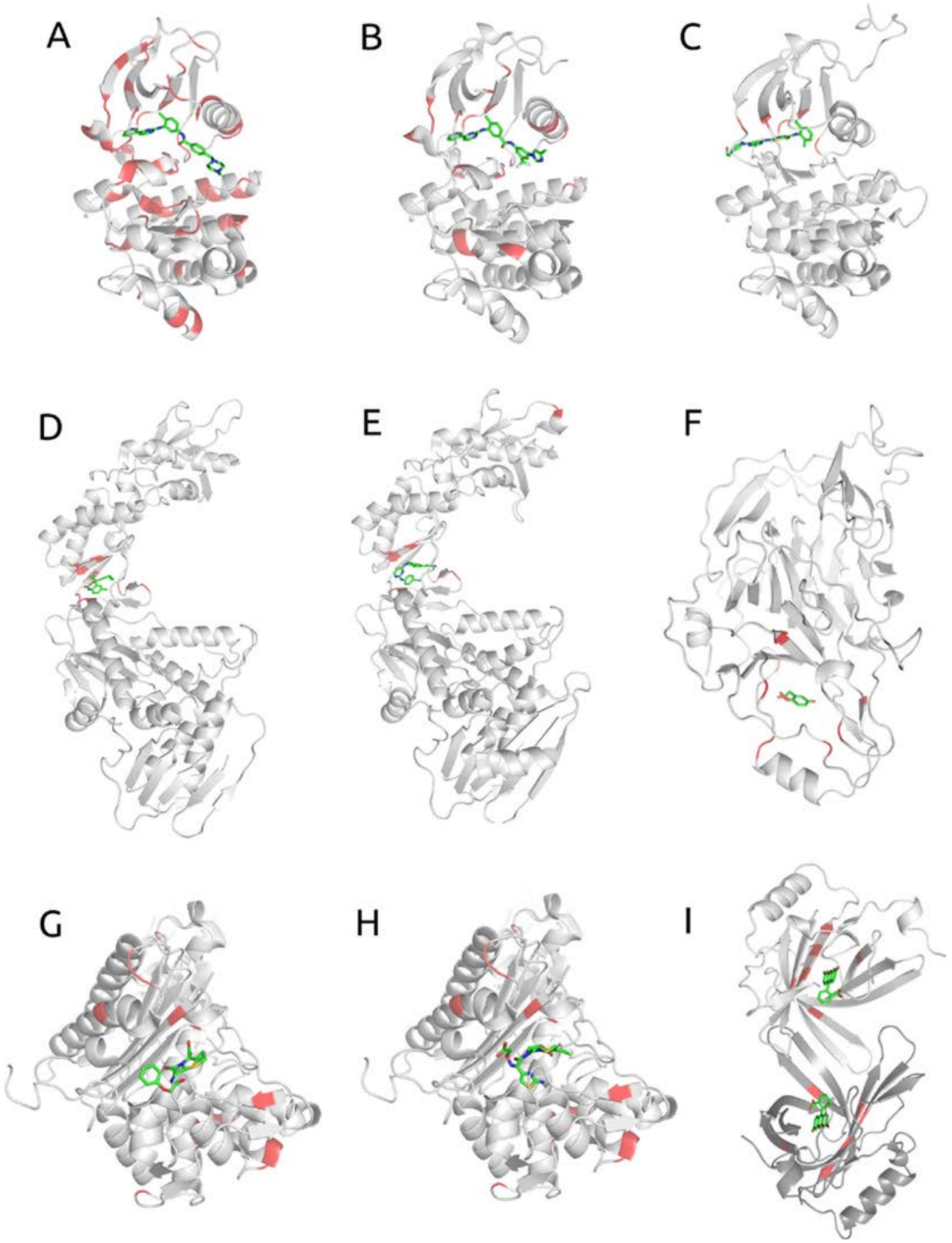

Figure S2. Spatial distribution of resistance and disease mutations in (A) ABL1-Imatinib; (B) ABL1-Nilotinib; (C) ABL1-Dasatinib; (D) HIV-RT-Efacirenz; (E) HIV-RT-Rilpivirine; (F) HGD-homogentisic acid; (G) *Neisseria gonorrhoeae* PBP2-Penicillin; (H) *Neisseria gonorrhoeae* PBP2-Cefixime; (I) FluA-Fluorescein complexes.

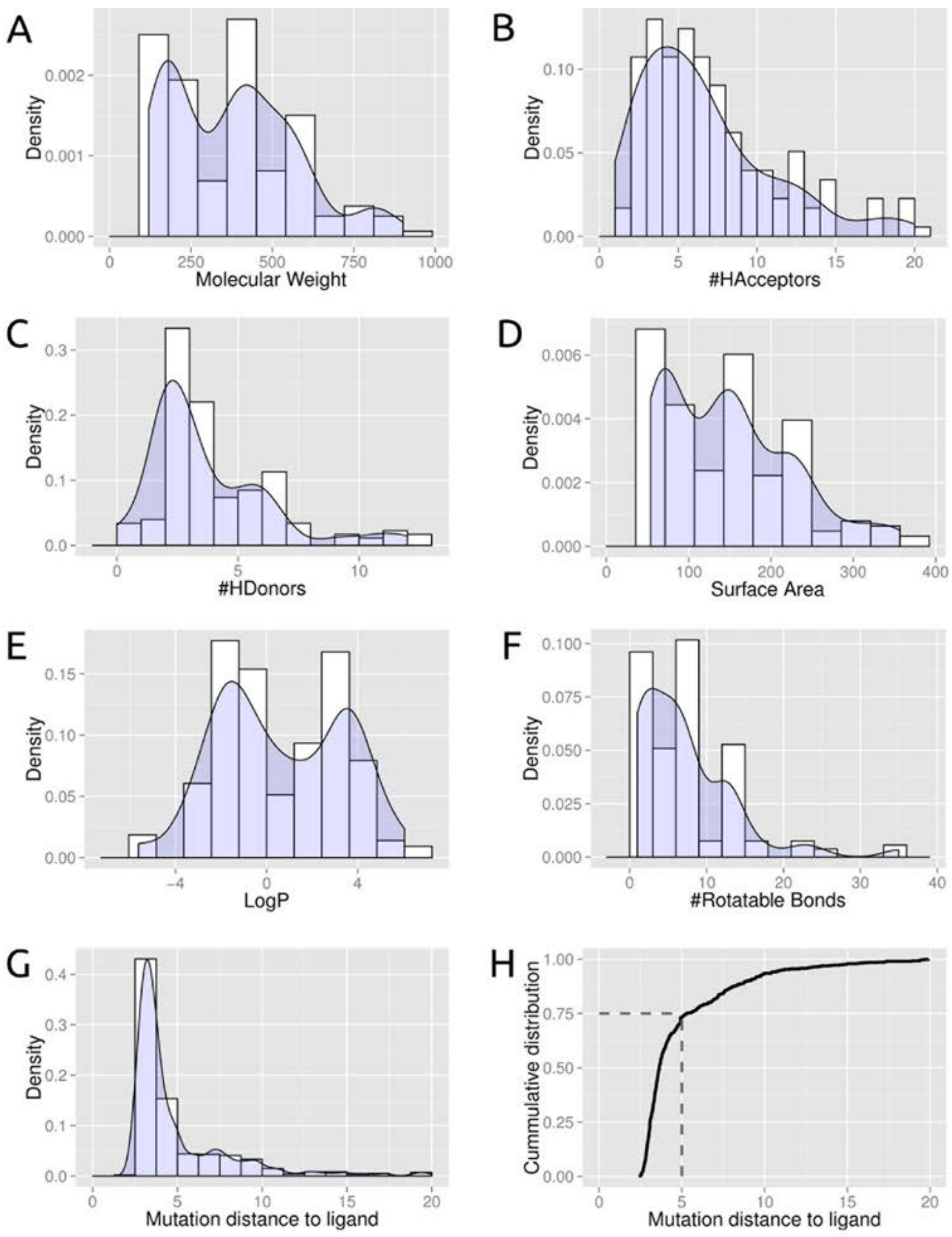

Figure S3. Histogram of molecular properties for the ligands present in the assembled dataset (177 unique ligands). (A) Molecular weight; (B) Hydrogen bond acceptors; (C) Hydrogen bond donors; (D) Total polar surface area; (E) LogP; and (F) Number of rotatable bonds. (G) Histogram and (H) cumulative distribution of the distances between mutated residues and ligands on the assembled dataset (763 mutations).

Supplementary Tables

Table S1. Performance of mCSM-lig under different validation procedures.

**Validation**	ρ	**Std. Error**
5-fold cross validation	0.612	2.087
10-fold cross validation	0.628	2.059
Leave-one-out cross validation	0.626	2.059
66% split	0.632	2.129

Table S2. Performance of the computational approach on classification tasks. The mCSM-lig denotes a combination of the described features and presents a significant improvement in performance in comparison with either individual feature. Performance assessed on 10-fold cross validation using a Random Forest.

**Feature**	**AUC**	**Precision**	**Accuracy**
Pharmacophore difference	0.595	0.644	0.666
Stability prediction	0.669	0.666	0.680
Ligand properties	0.722	0.680	0.688
Graph-based signatures	0.709	0.716	0.727
**mCSM-lig**	**0.791**	**0.755**	**0.761**

Table S3. Prediction of Imatinib, Nilotinib and Dasatinib resistance mutations. The table shows predictions for experimentally characterised mutations in BCR-ABL and the ratio between predictions of their effects in ATP and Imatinib, Nilotinib and Dasatinib binding.

**Mutation**	**Ligand**	**mCSM-lig Ligand**	**mCSM-lig ATP**	**Fold ratio Ligand-ATP**
A344V	Imatinib	-0.883	0.066	8.892
A350V	Imatinib	-0.626	0.306	8.551
A365V	Imatinib	-0.701	-0.352	2.234
A366G	Imatinib	-0.644	0.156	6.310
A380T	Imatinib	-1.711	-0.984	5.333
A397P	Imatinib	-0.172	-0.599	0.374
A433T	Imatinib	0.093	0.048	0.902
D276G	Imatinib	-1.007	0.432	27.479

D363Y	Imatinib	-0.634	-0.048	3.855
E255K	Imatinib	-1.057	-0.898	1.442
E255V	Imatinib	-1.348	0.716	115.878
E258D	Imatinib	-0.632	-0.263	2.339
E279K	Imatinib	-0.707	-0.158	3.540
E292Q	Imatinib	-0.173	-0.086	1.222
E292V	Imatinib	-0.516	0.621	13.709
E355A	Imatinib	0.534	0.358	0.667
E355D	Imatinib	-1.445	0.094	34.594
E355G	Imatinib	-1.46	-0.542	8.279
E373K	Imatinib	-0.511	0.318	6.745
E450A	Imatinib	-0.348	0.816	14.588
E450G	Imatinib	-1.743	0.227	93.325

E450K	Imatinib	-0.755	0.242	9.931
E450V	Imatinib	-1.612	-0.874	5.470
E453G	Imatinib	-0.83	0.143	9.397
E453K	Imatinib	-0.831	0.129	9.120
E453Q	Imatinib	-0.801	-1.022	0.601
E453V	Imatinib	-1.156	-1.092	1.159
E459G	Imatinib	-1.235	-1.018	1.648
E459K	Imatinib	-1.156	-0.982	1.493
E459Q	Imatinib	-0.982	-0.758	1.675
E459V	Imatinib	-0.544	-0.601	0.877
F311I	Imatinib	-0.477	-0.481	0.991
F311L	Imatinib	-1.115	-0.293	6.637
F317C	Imatinib	-0.419	-0.078	2.193

F317I	Imatinib	-1.072	-0.973	1.256
F317L	Imatinib	-0.602	-0.935	0.465
F317V	Imatinib	-0.188	-0.307	0.760
F359C	Imatinib	-1.171	0.139	20.417
F359I	Imatinib	-1.21	0.27	30.200
F359L	Imatinib	-1.109	0.215	21.086
F359V	Imatinib	-1.362	0.67	107.647
F382L	Imatinib	-1.321	0.442	57.943
F486S	Imatinib	-1.107	0.304	25.763
G250E	Imatinib	-1.322	0.463	60.954
G250R	Imatinib	-0.847	-0.199	4.446
I242T	Imatinib	-0.177	-0.039	1.374
I293V	Imatinib	-0.842	-0.386	2.858

I418S	Imatinib	-0.173	0.335	3.221
I418V	Imatinib	-0.861	-1.387	0.298
K247R	Imatinib	-0.627	-0.477	1.413
L248V	Imatinib	-0.569	0.454	10.544
L273M	Imatinib	0.511	-0.345	0.139
L298V	Imatinib	-2.145	-0.895	17.783
L324Q	Imatinib	-2.307	0.501	642.688
L364I	Imatinib	-1.194	0.072	18.450
L370P	Imatinib	0.29	0.632	2.198
L384M	Imatinib	0.37	0.638	1.854
L387F	Imatinib	0.344	0.889	3.508
L387M	Imatinib	-0.415	-0.147	1.854
L387V	Imatinib	-0.933	-0.467	2.924

M237V	Imatinib	0.008	-0.273	0.524
M244V	Imatinib	-0.247	0.185	2.704
M343T	Imatinib	-0.367	0.003	2.344
M351T	Imatinib	-0.567	0.006	3.741
M388L	Imatinib	-0.856	-0.01	7.015
M472I	Imatinib	-0.524	-0.375	1.409
P480L	Imatinib	0.302	0.336	1.081
S417F	Imatinib	-0.298	0.576	7.482
S417Y	Imatinib	-0.168	0.664	6.792
S438C	Imatinib	-0.103	0.603	5.082
T277A	Imatinib	-0.112	0.485	3.954
T315A	Imatinib	-0.028	0.788	6.546
T315I	Imatinib	-0.3	0.72	10.471

V280A	Imatinib	-0.013	0.411	2.655
V289A	Imatinib	0.144	1.089	8.810
V289I	Imatinib	-0.265	0.386	4.477
V299L	Imatinib	-0.085	0.378	2.904
V371A	Imatinib	-0.028	0.578	4.036
V379I	Imatinib	0.06	0.718	4.550
W261L	Imatinib	-0.957	-0.957	1.000
Y320C	Imatinib	-0.923	-0.383	3.467
Y342H	Imatinib	0.642	0.85	1.614
T315I	NIlotinib	0.468	-1.022	0.032
E255V	NIlotinib	-0.87	-0.048	6.637
L248V	NIlotinib	-1.675	-0.984	4.909
K285N	NIlotinib	-1.079	0.041	13.183

E282K	NIlotinib	-1.391	0.488	75.683
E255K	NIlotinib	-1.377	0.432	64.417
F359C	NIlotinib	-1.251	0.304	35.892
F359V	NIlotinib	-1.483	0.67	142.233
K247N	NIlotinib	-0.271	-0.161	1.288
W430L	NIlotinib	-0.705	0.76	29.174
T406I	NIlotinib	-0.684	-0.71	0.942
E255R	NIlotinib	-1.723	0.056	60.117
A380S	NIlotinib	-1.548	-0.777	5.902
F311V	NIlotinib	-1.178	0.352	33.884
L273F	NIlotinib	-0.948	-0.915	1.079
H375P	NIlotinib	-0.361	0.085	2.793
E292K	NIlotinib	-0.756	0.416	14.859

L387M	NIlotinib	0.035	0.632	3.954
G250E	NIlotinib	-0.258	-0.599	0.456
E431G	NIlotinib	-1.002	-0.492	3.236
V379I	NIlotinib	0.222	-0.345	0.271
F317L	NIlotinib	-1.468	-1.092	2.377
F311L	NIlotinib	-1.059	0.143	15.922
N297T	NIlotinib	-0.769	0.35	13.152
T315I	Dasatinib	0.964	-1.022	0.010
T315A	Dasatinib	0.25	-1.007	0.055
F317V	Dasatinib	-2.493	-1.018	29.854
V299L	Dasatinib	-0.383	-0.874	0.323
L248R	Dasatinib	-1.895	-0.748	14.028
F317L	Dasatinib	-2.334	-1.092	17.458

E255V	Dasatinib	-1.09	-0.048	11.015
E255K	Dasatinib	-1.736	0.432	147.231

Table S4. Prediction of HIV-RT resistance mutations. The table shows predictions for mutations on the HIV-RT/Rilpivirine and HIV-RT/Efavirenz complexes.

**Drug**	**Mutation**	**mCSM-lig**
Rilpivirine	E138A	-0.425
Rilpivirine	E138G	-0.429
Rilpivirine	E138K	-0.416
Rilpivirine	E138Q	-0.295
Rilpivirine	E138R	-0.481
Rilpivirine	F227C	-1.409
Rilpivirine	G190A	-0.402
Rilpivirine	G190E	-1.317
Rilpivirine	G190Q	-0.949
Rilpivirine	G190S	-0.862
Rilpivirine	H221Y	0.092

Rilpivirine	K101E	-0.152
Rilpivirine	K101H	-0.929
Rilpivirine	K101P	0.772
Rilpivirine	L100I	-1.923
Rilpivirine	M230I	-0.425
Rilpivirine	M230L	-0.407
Rilpivirine	V179D	-0.528
Rilpivirine	V179E	-0.501
Rilpivirine	V179F	-0.067
Rilpivirine	V179L	-0.425
Rilpivirine	Y181C	-1.215
Rilpivirine	Y181I	-1.458
Rilpivirine	Y181V	-1.688
Rilpivirine	Y188L	-1.433
Efavirenz	F227C	-1.612

Efavirenz	F227L	-1.955
Efavirenz	G190A	-0.21
Efavirenz	G190E	-1.161
Efavirenz	G190Q	-0.357
Efavirenz	G190S	-0.36
Efavirenz	K101E	-0.392
Efavirenz	K101H	-0.586
Efavirenz	K101P	0.373
Efavirenz	K103N	-0.636
Efavirenz	K103S	-1.093
Efavirenz	L100I	-0.364
Efavirenz	M230L	-0.217
Efavirenz	P225H	-0.891
Efavirenz	V106A	-1.586
Efavirenz	V106M	-0.806

Efavirenz	V179D	-0.508
Efavirenz	V179E	-0.483
Efavirenz	V179F	-0.085
Efavirenz	Y181C	-1.461
Efavirenz	Y188C	-0.849
Efavirenz	Y188H	-1.268
Efavirenz	Y188L	-0.408

Table S5. Prediction of effects of mutations on ligand-affinity in Alcaptonuria and Miller's Syndrome. The table shows mCMS-lig predictions for mutations on the HGD/Homogentisic acid (LIGID: OMD) complex and the DHODH-ligand complexes.

Protein	Ligand ID	Mutation	Phenotype	mCSM-lig
HGD	OMD	C120W	Pathogenic	-1.089
HGD	OMD	E401Q	Pathogenic	-2.835
HGD	OMD	G161R	Pathogenic	-0.806
HGD	OMD	G270R	Pathogenic	-0.501
HGD	OMD	H371R	Pathogenic	-0.053
HGD	OMD	M368V	Pathogenic	-0.103
HGD	OMD	N337D	Pathogenic	-0.246
HGD	OMD	P230S	Pathogenic	0.15
HGD	OMD	R321P	Pathogenic	-1.813
HGD	OMD	R330S	Pathogenic	-2.28
HGD	OMD	S47L	Pathogenic	-0.547

HGD	OMD	V300G	Pathogenic	-0.895
HGD	OMD	A122V	Non-Pathogenic	-1.005
HGD	OMD	A267D	Non-Pathogenic	0.077
HGD	OMD	A293E	Non-Pathogenic	1.111
HGD	OMD	A381P	Non-Pathogenic	-0.337
HGD	OMD	A392T	Non-Pathogenic	0.651
HGD	OMD	A397T	Non-Pathogenic	-0.05
HGD	OMD	A48S	Non-Pathogenic	0.693
HGD	OMD	A48T	Non-Pathogenic	0.687
HGD	OMD	D153N	Non-Pathogenic	-2.451
HGD	OMD	D212N	Non-Pathogenic	-0.006
HGD	OMD	D326N	Non-Pathogenic	-0.341
HGD	OMD	D376E	Non-Pathogenic	0.343
HGD	OMD	E101V	Non-Pathogenic	-0.285
HGD	OMD	E168K	Non-Pathogenic	-1.76

HGD	OMD	E379Q	Non-Pathogenic	-0.423
HGD	OMD	E3A	Non-Pathogenic	-0.329
HGD	OMD	E42A	Non-Pathogenic	-1.027
HGD	OMD	E74V	Non-Pathogenic	-0.235
HGD	OMD	E87A	Non-Pathogenic	-0.605
HGD	OMD	F10L	Non-Pathogenic	-0.017
HGD	OMD	F282V	Non-Pathogenic	-0.401
HGD	OMD	G11E	Non-Pathogenic	1.173
HGD	OMD	G123E	Non-Pathogenic	0.781
HGD	OMD	G123R	Non-Pathogenic	-0.514
HGD	OMD	G185E	Non-Pathogenic	0.306
HGD	OMD	G185V	Non-Pathogenic	-0.587
HGD	OMD	G198D	Non-Pathogenic	-0.679
HGD	OMD	G205S	Non-Pathogenic	0.608
HGD	OMD	G23D	Non-Pathogenic	0.944

HGD	OMD	G360R	Non-Pathogenic	-0.745
HGD	OMD	G361E	Non-Pathogenic	-0.796
HGD	OMD	G362E	Non-Pathogenic	-0.768
HGD	OMD	G394C	Non-Pathogenic	0.212
HGD	OMD	H207Y	Non-Pathogenic	-0.863
HGD	OMD	I133V	Non-Pathogenic	-1.248
HGD	OMD	I231T	Non-Pathogenic	0.666
HGD	OMD	I246V	Non-Pathogenic	-0.358
HGD	OMD	K327E	Non-Pathogenic	0.115
HGD	OMD	K414N	Non-Pathogenic	0.403
HGD	OMD	L116P	Non-Pathogenic	0.066
HGD	OMD	L163F	Non-Pathogenic	-0.428
HGD	OMD	L279M	Non-Pathogenic	-0.933
HGD	OMD	L386P	Non-Pathogenic	0.198
HGD	OMD	L413F	Non-Pathogenic	0.467

HGD	OMD	M339I	Non-Pathogenic	-0.779
HGD	OMD	M396I	Non-Pathogenic	-0.728
HGD	OMD	N177S	Non-Pathogenic	-1.277
HGD	OMD	N271I	Non-Pathogenic	-1.285
HGD	OMD	N278D	Non-Pathogenic	-1.126
HGD	OMD	N286K	Non-Pathogenic	-0.499
HGD	OMD	N440K	Non-Pathogenic	-0.595
HGD	OMD	N56D	Non-Pathogenic	0.148
HGD	OMD	N93K	Non-Pathogenic	0.077
HGD	OMD	P103T	Non-Pathogenic	0.574
HGD	OMD	P158L	Non-Pathogenic	-1.612
HGD	OMD	P224S	Non-Pathogenic	1.152
HGD	OMD	P308R	Non-Pathogenic	0.188
HGD	OMD	P32L	Non-Pathogenic	0.641
HGD	OMD	P373L	Non-Pathogenic	-0.361

HGD	OMD	P436L	Non-Pathogenic	-0.135
HGD	OMD	Q258L	Non-Pathogenic	-1.314
HGD	OMD	R20H	Non-Pathogenic	0.189
HGD	OMD	R225P	Non-Pathogenic	-0.609
HGD	OMD	R307C	Non-Pathogenic	-0.818
HGD	OMD	R307H	Non-Pathogenic	-0.208
HGD	OMD	R53Q	Non-Pathogenic	-0.366
HGD	OMD	R58I	Non-Pathogenic	-1.328
HGD	OMD	S106F	Non-Pathogenic	-0.18
HGD	OMD	S114R	Non-Pathogenic	-0.691
HGD	OMD	S114T	Non-Pathogenic	0.106
HGD	OMD	S24F	Non-Pathogenic	-0.386
HGD	OMD	S47L	Non-Pathogenic	-0.547
HGD	OMD	S54N	Non-Pathogenic	0.275
HGD	OMD	S67P	Non-Pathogenic	-0.433

HGD	OMD	T140A	Non-Pathogenic	-1.183
HGD	OMD	T244A	Non-Pathogenic	-1.015
HGD	OMD	T299A	Non-Pathogenic	-1.13
HGD	OMD	T302N	Non-Pathogenic	-0.573
HGD	OMD	T409S	Non-Pathogenic	-0.316
HGD	OMD	V181I	Non-Pathogenic	-1.398
HGD	OMD	V245A	Non-Pathogenic	-0.028
HGD	OMD	V265A	Non-Pathogenic	-0.053
HGD	OMD	V316F	Non-Pathogenic	-1.296
HGD	OMD	V81G	Non-Pathogenic	0.098
HGD	OMD	W233R	Non-Pathogenic	-0.26
HGD	OMD	Y166C	Non-Pathogenic	-0.337
DHODH	FMN	Pathogenic	G153R	-1.335
DHODH	FMN	Pathogenic	G203A	-0.282
DHODH	FMN	Pathogenic	G203D	0.18

DHODH	FMN	Pathogenic	R136C	-0.36
DHODH	FMN	Pathogenic	R200C	-0.183
DHODH	FMN	Pathogenic	R245W	-0.087
DHODH	FMN	Pathogenic	R246W	-0.23
DHODH	FMN	Pathogenic	R347W	-0.02
DHODH	FMN	Pathogenic	T286I	0.994
DHODH	ORO	Pathogenic	G153R	-1.031
DHODH	ORO	Pathogenic	G203A	-1.32
DHODH	ORO	Pathogenic	G203D	-0.092
DHODH	ORO	Pathogenic	R136C	-1.749
DHODH	ORO	Pathogenic	R200C	-1.393
DHODH	ORO	Pathogenic	R245W	-0.274
DHODH	ORO	Pathogenic	R246W	-0.363
DHODH	ORO	Pathogenic	R347W	-0.813
DHODH	ORO	Pathogenic	T286I	-2.011

DHODH	FMN	Non-Pathogenic	A192V	-0.343
DHODH	FMN	Non-Pathogenic	A196T	0.169
DHODH	FMN	Non-Pathogenic	A291V	0.246
DHODH	FMN	Non-Pathogenic	A296V	1.093
DHODH	FMN	Non-Pathogenic	A322S	0.879
DHODH	FMN	Non-Pathogenic	A342V	0.069
DHODH	FMN	Non-Pathogenic	A348V	0.532
DHODH	FMN	Non-Pathogenic	D106Y	0.329
DHODH	FMN	Non-Pathogenic	D278N	0.515
DHODH	FMN	Non-Pathogenic	E229K	0.811
DHODH	FMN	Non-Pathogenic	E274K	0.759
DHODH	FMN	Non-Pathogenic	E35D	0.471
DHODH	FMN	Non-Pathogenic	E380A	0.522
DHODH	FMN	Non-Pathogenic	E53Q	0.174
DHODH	FMN	Non-Pathogenic	F361L	-0.471

DHODH	FMN	Non-Pathogenic	G107R	-1.923
DHODH	FMN	Non-Pathogenic	G220R	0.383
DHODH	FMN	Non-Pathogenic	G295D	0.573
DHODH	FMN	Non-Pathogenic	G385R	-1.483
DHODH	FMN	Non-Pathogenic	G48E	-0.072
DHODH	FMN	Non-Pathogenic	H248R	0.382
DHODH	FMN	Non-Pathogenic	I118L	0.099
DHODH	FMN	Non-Pathogenic	I268L	0.099
DHODH	FMN	Non-Pathogenic	L154I	-0.062
DHODH	FMN	Non-Pathogenic	L352M	-1.275
DHODH	FMN	Non-Pathogenic	L355V	-0.75
DHODH	FMN	Non-Pathogenic	L65M	0.906
DHODH	FMN	Non-Pathogenic	L84M	0.393
DHODH	FMN	Non-Pathogenic	P329L	-0.511
DHODH	FMN	Non-Pathogenic	P44L	0.585

DHODH	FMN	Non-Pathogenic	P52L	-0.784
DHODH	FMN	Non-Pathogenic	R136H	-0.052
DHODH	FMN	Non-Pathogenic	R162Q	0.138
DHODH	FMN	Non-Pathogenic	R222Q	-0.381
DHODH	FMN	Non-Pathogenic	R231C	-0.196
DHODH	FMN	Non-Pathogenic	R232C	-0.005
DHODH	FMN	Non-Pathogenic	R298C	-0.038
DHODH	FMN	Non-Pathogenic	R298H	0.704
DHODH	FMN	Non-Pathogenic	R318W	0.368
DHODH	FMN	Non-Pathogenic	R36C	-0.085
DHODH	FMN	Non-Pathogenic	R36H	0.866
DHODH	FMN	Non-Pathogenic	R372Q	-0.058
DHODH	FMN	Non-Pathogenic	R372W	0.17
DHODH	FMN	Non-Pathogenic	R57H	0.535
DHODH	FMN	Non-Pathogenic	R61H	0.736

DHODH	FMN	Non-Pathogenic	R70Q	-0.34
DHODH	FMN	Non-Pathogenic	R70W	-0.241
DHODH	FMN	Non-Pathogenic	S288G	0.383
DHODH	FMN	Non-Pathogenic	S338C	-0.238
DHODH	FMN	Non-Pathogenic	T357M	-0.42
DHODH	FMN	Non-Pathogenic	V105M	-0.296
DHODH	FMN	Non-Pathogenic	V199M	-0.357
DHODH	FMN	Non-Pathogenic	V201I	-0.128
DHODH	FMN	Non-Pathogenic	V254A	-0.521
DHODH	FMN	Non-Pathogenic	V272I	-0.361
DHODH	FMN	Non-Pathogenic	V60G	0.236
DHODH	FMN	Non-Pathogenic	W362C	0.102
DHODH	ORO	Non-Pathogenic	A192V	-1.177
DHODH	ORO	Non-Pathogenic	A196T	0.354
DHODH	ORO	Non-Pathogenic	A291V	-0.035

DHODH	ORO	Non-Pathogenic	A296V	0.312
DHODH	ORO	Non-Pathogenic	A322S	0.905
DHODH	ORO	Non-Pathogenic	A342V	-0.653
DHODH	ORO	Non-Pathogenic	A348V	-0.677
DHODH	ORO	Non-Pathogenic	D106Y	-0.694
DHODH	ORO	Non-Pathogenic	D278N	-0.781
DHODH	ORO	Non-Pathogenic	E229K	-0.3
DHODH	ORO	Non-Pathogenic	E274K	-0.081
DHODH	ORO	Non-Pathogenic	E35D	-0.292
DHODH	ORO	Non-Pathogenic	E380A	-0.284
DHODH	ORO	Non-Pathogenic	E53Q	-1.87
DHODH	ORO	Non-Pathogenic	F361L	-1.929
DHODH	ORO	Non-Pathogenic	G107R	-0.857
DHODH	ORO	Non-Pathogenic	G220R	0.595
DHODH	ORO	Non-Pathogenic	G295D	1.759

DHODH	ORO	Non-Pathogenic	G385R	-0.309
DHODH	ORO	Non-Pathogenic	G48E	0.882
DHODH	ORO	Non-Pathogenic	H248R	0.518
DHODH	ORO	Non-Pathogenic	I118L	-0.727
DHODH	ORO	Non-Pathogenic	I268L	-0.643
DHODH	ORO	Non-Pathogenic	L154I	-1.22
DHODH	ORO	Non-Pathogenic	L352M	-1.556
DHODH	ORO	Non-Pathogenic	L355V	-0.641
DHODH	ORO	Non-Pathogenic	L65M	0.148
DHODH	ORO	Non-Pathogenic	L84M	-0.137
DHODH	ORO	Non-Pathogenic	P329L	-0.907
DHODH	ORO	Non-Pathogenic	P44L	-0.066
DHODH	ORO	Non-Pathogenic	P52L	-1.514
DHODH	ORO	Non-Pathogenic	R136H	-1.366
DHODH	ORO	Non-Pathogenic	R162Q	-0.838

DHODH	ORO	Non-Pathogenic	R222Q	-1.556
DHODH	ORO	Non-Pathogenic	R231C	-1.338
DHODH	ORO	Non-Pathogenic	R232C	-0.996
DHODH	ORO	Non-Pathogenic	R298C	-1.007
DHODH	ORO	Non-Pathogenic	R298H	-0.304
DHODH	ORO	Non-Pathogenic	R318W	-0.638
DHODH	ORO	Non-Pathogenic	R36C	-0.614
DHODH	ORO	Non-Pathogenic	R36H	0.123
DHODH	ORO	Non-Pathogenic	R372Q	-0.859
DHODH	ORO	Non-Pathogenic	R372W	-0.469
DHODH	ORO	Non-Pathogenic	R57H	-0.429
DHODH	ORO	Non-Pathogenic	R61H	-0.094
DHODH	ORO	Non-Pathogenic	R70Q	-0.402
DHODH	ORO	Non-Pathogenic	R70W	-0.342
DHODH	ORO	Non-Pathogenic	S288G	-0.883

DHODH	ORO	Non-Pathogenic	S338C	-0.972
DHODH	ORO	Non-Pathogenic	T357M	-1.428
DHODH	ORO	Non-Pathogenic	V105M	-0.879
DHODH	ORO	Non-Pathogenic	V199M	-1.425
DHODH	ORO	Non-Pathogenic	V201I	-0.723
DHODH	ORO	Non-Pathogenic	V254A	-0.537
DHODH	ORO	Non-Pathogenic	V272I	-1.155
DHODH	ORO	Non-Pathogenic	V60G	-0.41
DHODH	ORO	Non-Pathogenic	W362C	-0.879

Table S6. Prediction of effects of mutations on Penicillin and Cefixime-affinity on *N. gonorrhoeae* PBP2. The table shows mCMS-lig predictions for mutations on the PBP2/Penicillin and Cefixime complexes.

**Mutation**	**Penicillin**	**Cefixime**
A376P	-0.301	-0.828
A376S	0.316	-0.301
A501P	-0.765	0.287
A501T	-0.147	0.156
A501V	-0.74	0.19
A516G	0.123	0.513
D285E	0.348	0.49
D285N	-0.162	0.475
E385D	1.261	1.565
E385S	0.512	1.296
G375S	0.791	0.243
G375T	0.723	0.151

G542S	0.344	0.305
H541N	0.225	0.045
H541Y	0.554	0.424
I566V	1.163	1.491
L328A	1.166	1.636
L328T	1.346	1.202
P343Q	0.902	0.56
P343T	1.039	0.805
P551S	0.161	-0.168
V316P	-0.888	-1.418

.

References

1. Pires DE, *et al.* (2011) Cutoff Scanning Matrix (CSM): structural classification and function prediction by protein inter-residue distance patterns. *BMC Genomics* 12 Suppl 4:S12.
2. Pires DE, Ascher DB, & Blundell TL (2014) mCSM: predicting the effects of mutations in proteins using graph-based signatures. *Bioinformatics* 30(3):335-342.
3. Yamachika S, Sugihara C, Kamai Y, & Yamashita M (2013) Correlation between penicillin-binding protein 2 mutations and carbapenem resistance in Escherichia coli. *J Med Microbiol* 62(Pt 3):429-436.
4. Beste G, Schmidt FS, Stibora T, & Skerra A (1999) Small antibody-like proteins with prescribed ligand specificities derived from the lipocalin fold. *Proc Natl Acad Sci U S A* 96(5):1898-1903.
5. Vopel S, Muhlbach H, & Skerra A (2005) Rational engineering of a fluorescein-binding anticalin for improved ligand affinity. *Biol Chem* 386(11):1097-1104.
